# Left Atrial Appendage Closure to Prevent Strokes and Ligation to Prevent Atrial Fibrillation

**DOI:** 10.19102/icrm.2017.081204

**Published:** 2017-12-15

**Authors:** Christopher R. Ellis

**Affiliations:** ^1^Vanderbilt Heart and Vascular Institute, Nashville, TN, USA

**Keywords:** Anticoagulation, atrial fibrillation, left atrial appendage, stroke

In the two years following United States Food and Drug Administration (FDA) approval of the first left atrial appendage (LAA) closure device for the prevention of embolic stroke in patients with nonvalvular atrial fibrillation (AF), over 6000 WATCHMAN^™^ (Boston Scientific, Inc., Natick, MA, USA) devices have been successfully implanted nationwide. Postapproval prospective registry data of 3,822 consecutive cases demonstrated that device implantation was successful in 3,653 (95.6%) with a median procedure time of 50 minutes, despite most of the operators being new to the procedure (71%). Complications included 39 pericardial tamponades (1.02%), three instances of procedure-related stroke (0.078%), nine device embolizations (0.24%), and three procedure-related deaths (0.078%).^1^ Though complications in early phase studies such as PROTECT-AF exceeded 10%, as expertise in transseptal access has evolved and improvements in the delivery system and implantation technique have been made, total complications postimplant have dropped to <3%, as noted in the EWOLUTION registry.^2^

With proper patient selection, routine use of ultrasound to guide venous access, and a continued trend to implant in conjunction with uninterrupted anticoagulation, expert centers should be able to push total complications below 1%. As a stroke prevention strategy, this could position LAA closure as a cost-effective consideration, and potentially as first-line therapy for many patients. Critics rightfully remark that there is a paucity of prospective randomized data outside of those from the PROTECT-AF and PREVAIL trials, and that the total number of stroke or embolic events under study is extremely small. Additionally, the determination of what is an acceptable LAA anatomy to close with a WATCHMAN^™^ device (Boston Scientific, Inc., Natick, MA, USA) is quite subjective. This has introduced significant selection bias into clinical trials of endocardial occlusion devices and may limit the generalizability of a “98%” implant success for the WATCHMAN^™^ (Boston Scientific, Inc., Natick, MA, USA) device specifically.

In general, LAA closure is gathering awareness and acceptance, with three critical questions in existence that warrant careful study heading into 2018: first, do devices or approaches other than the WATCHMAN^™^ (Boston Scientific, Inc., Natick, MA, USA) truly protect from embolic AF related stroke? Second, what does the presence of leaks around a device or device-associated thrombus (DAT) mean? Lastly, does the removal of the LAA impact arrhythmia recurrence after catheter or surgical ablation?

## Eyeing the future

Several landmark clinical trials ongoing in the LAA occlusion space are expected to provide prospective randomized trial data on over 5,000 patients closed with a variety of methods to help answer these questions. A randomized trial (NCT02879448) pitting the AMPLATZER^™^ Amulet^™^ (Abbott Laboratories, Chicago, IL, USA) against the WATCHMAN^™^ (Boston Scientific, Inc., Natick, MA, USA) device has been enrolling participants since mid-2017, with a target enrollment of 1400 subjects and 300 roll-in cases. Patients eligible to receive a WATCHMAN^™^ LAA closure device (Boston Scientific, Inc., Natick, MA, USA) will be randomized 1:1 to receive either an AMPLATZER^™^ Amulet^™^ (Abbott Laboratories, Chicago, IL, USA) or a WATCHMAN^™^ (Boston Scientific, Inc., Natick, MA, USA) device upon enrollment and followed for a minimum of 18 months for the endpoint of stroke or systemic embolism. Anticoagulation regimen and antiplatelet therapy postimplant will be done according to the FDA labeling for patients who receive the WATCHMAN^™^ (Boston Scientific, Inc., Natick, MA, USA), with a more variable strategy for the AMPLATZER^™^ Amulet^™^ (Abbott Laboratories, Chicago, IL, USA) arm, including even dual antiplatelet therapy alone (aspirin 81 mg plus clopidogrel 75 mg for a minimum of six months, followed by aspirin 81 mg alone). Given the current enrollment rate (per Abbott Medical, 728 subjects have been enrolled as of October 5, 2017), the trial will likely complete enrollment in 2018.

The Left Atrial Appendage Occlusion Study III (LAAOS III) is a multinational clinical prospective randomized trial of surgical LAA closure (done by stapled excision or surgical excision) at the time of open heart surgery in patients with a history of AF.^3^ The study will enroll 4,700 patients with a five-year clinical follow up period; to date, over 3,500 subjects have enrolled offering data from more than 130 clinical events. This is nearly a 10-fold higher rate of primary clinical endpoints than that seen in the clinical trials involving the WATCHMAN^™^ (Boston Scientific, Inc., Natick, MA, USA) LAA closure device. Interestingly, there is no anticoagulation regimen protocol in the LAAOS III, which may impact the final analysis. The study could provide sufficient data to ultimately support a protective effect of removing the LAA independent of the use of anticoagulation, but this remains to be seen at its conclusion.

Referrals for LAA closure for anticoagulation failure (characterized by thrombus formation in the LAA despite medical therapy or recurrent embolic events/stroke while on oral anticoagulation) are not clearly outlined in any existing LAA closure clinical trial inclusion criteria. In our experience, these may actually be the patients who stand to benefit the most from removing or occluding the LAA. The lack of use of the AtriClip^®^ or AtriClip^®^ PRO system (both AtriCure, Inc. West Chester, OH, USA) for LAA closure in the LAAOS III is disappointing, particularly given that two separate studies reported a more than 93% LAA closure efficacy rate, including in a 2017 LAA closure study involving long-term computed tomography (CT) angiography assessment with the use of the AtriClip^®^ (AtriCure, Inc. West Chester, OH, USA) placed by thoracoscopic approach.^4,5^ Other prior formal studies of surgical methods have yielded 60% to 70% closure rates at best, and incomplete surgical closure is associated with a higher rate of stroke.^6^

**Figure 1:**
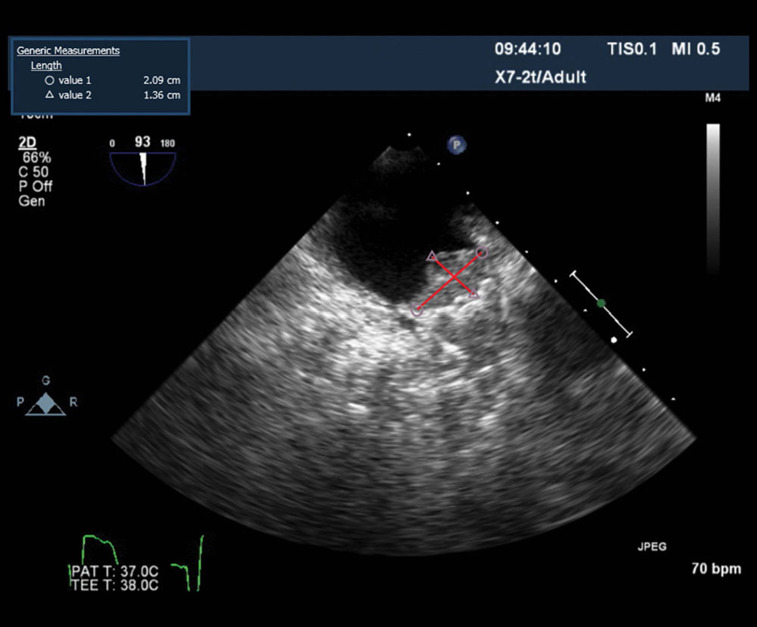
An example of DAT.

Several retrospective studies were published in 2017 supporting the use of novel oral anticoagulation (NOAC) or direct oral anticoagulation (DOAC) for anticoagulation post LAA device implantation to reduce DAT **(Figure 1)**. EWOLUTION reported a one-year device-associated thrombus rate of 3.7% with WATCHMAN^™^ (Boston Scientific, Inc., Natick, MA, USA), independent of the postimplant regimen, in which 73% of anticoagulation-contraindicated subjects were on antiplatelet regimens alone.^2^ A separate study showed a 3.2% DAT rate with the AMPLATZER^™^ Cardiac Plug, a previous generation of the AMPLATZER^™^ Amulet^™^ (both Abbott Laboratories, Chicago, IL, USA) currently under investigational device exemption (IDE) trial in the US.^7^ While it is certainly not what the implanting physician hopes to see at the 45-day or one-year follow-up on transesophageal echocardiogram (TEE), whether DAT leads to clinical ischemic strokes as a whole has been difficult to determine. Our practice has been to initiate short-term oral anticoagulation and to repeat the TEE in six weeks to ensure resolution, and then return the patient to using antiplatelet therapy alone. Several trials are currently being designed to prospectively assess the utility of NOAC or DOAC therapy post-LAA closure in hopes of reducing DAT prevalence.

The occurrence of peridevice leaks around a LAA occluder is also an evolving story, with the potential for an associated risk of late ischemic strokes, and there is little evidence of what to do in the setting of an edge leak in conjunction with an LAA closure device. Results from a multicenter registry using the AMPLATZER^™^ Cardiac Plug (Abbott Laboratories, Chicago, IL, USA) showed a 12.5% rate of leak around the device with TEE at a median of four months, which was not associated with an increased risk for stroke at one year.^8^ However, a separate study completed in 2017 using CT angiography demonstrated contrast flow across 66% of LAA devices at six weeks, questioning if a TEE performed at that time point is really a suitable metric for determining adequate closure.^8^ Likely, this represents the occurrence of a slow leak through the fabric cap and not around the device, if the TEE shows no Doppler flow. We have seen several cases in which follow-up TEE missed a leak and an obvious gap with contrast filling and a trabeculated LAA lobe was seen on CT angiography. Anticoagulation was discontinued in these patients, but long-term research is needed to evaluate the impact of this finding.

**Figure 2:**
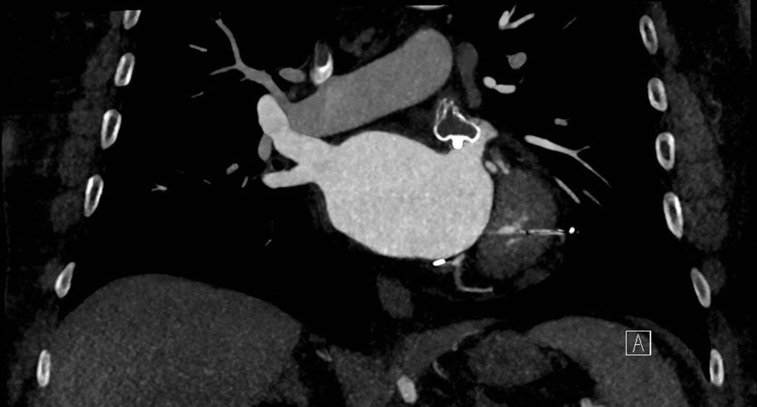
A CT scan demonstrating a large leak around a WATCHMAN^™^ (Boston Scientific, Inc., Natick, MA, USA) device.

The desire to implant a highly compressed WATCHMAN^™^ (Boston Scientific, Inc., Natick, MA, USA) device to reduce the chance for an edge leak is most relevant at the extremes of device sizes **(Figure 2)**. A recent study showed that LAA ostia smaller than those specified in the directions for use (<17 mm) for WATCHMAN^™^ (Boston Scientific, Inc., Natick, MA, USA) can be closed effectively, though caution is advised in these cases to ensure good compression, and abstaining from undersizing a smaller device to avoid embolization is suggested.^9^ In our experience with LAA ostia measuring closer to 31 mm, closure can still be accomplished if at least two implant angles on TEE are <28 mm, and 8% to 10% compression is clearly seen with a stable tug test on a 33-mm WATCHMAN^™^ (Boston Scientific, Inc., Natick, MA, USA) device. We make an extra effort to avoid any TEE or contrast leak at implant, with the ultimate goal of obliteration of any trabeculated portion of the LAA.

Finally, an ongoing IDE trial of the LARIAT^®^ system (SentreHEART, Redwood City, CA, USA) for ligation of the LAA as an adjunct to catheter ablation for persistent or longstanding persistent AF has reached the point of the FDA conducting an important safety endpoint review. The aMAZE study has also enrolled over 200 subjects to date, with an interim analysis possible after 400 patients are enrolled and treated.^10^ If LAA ligation effectively improves the outcome after catheter ablation for AF, it would provide a much more appealing option for clinicians to consider in patient treatment than the act of simply electrically isolating the LAA with a catheter. Electrical isolation without mechanical closure could increase the risk for thrombus in the LAA and stroke, even in sinus rhythm.^11^ This would effectively necessitate indefinite anticoagulation, which is something many believe we should never do. Whether LAA removal electrically truly improves ablation success is controversial. The BELIEF trial supports this concept, demonstrating a 20% improvement in AF-free survival at one year in patients with persistent AF who underwent extensive catheter ablation; however, a hybrid AF surgical ablation trial with or without LAA removal did not show any improvement in arrhythmia-free survival at 18 months.^12,13^ The aMAZE trial may provide the ultimate verdict in the end, as the ablation set used is simply pulmonary vein isolation and a right-sided typical flutter ablation line is present in all subjects, with adjunctive LARIAT^®^ (SentreHEART, Redwood City, CA, USA) use in a 2:1 fashion.

In conclusion, the table has been set for three landmark clinical trials to potentially complete enrollment in 2018, and these could establish a much broader application for LAA closure in patients with AF. Regardless of these studies’ findings, it is imperative that both physicians and industry partners provide this therapy to the appropriate patients with the highest level of safety, and closure efficacy. Until more data are collected, we must carefully consider using these devices in our patients.
